# Outcomes when congenital heart disease is diagnosed antenatally versus postnatally in the UK: a retrospective population-based study

**DOI:** 10.1186/s12887-015-0370-3

**Published:** 2015-05-16

**Authors:** Lewis K Peake, Elizabeth S Draper, Judith LS Budd, David Field

**Affiliations:** Department of Health Sciences, University of Leicester, Leicester, LE1 6TP UK

**Keywords:** Heart defects, Congenital, Transposition of great vessels, Hypoplastic left heart syndrome, Prenatal diagnosis

## Abstract

**Background:**

For major congenital heart disease, the benefits of antenatal diagnosis on some post-natal measures have been suggested. However, findings have been inconclusive and focus on short term outcome measures alone with little data from a UK population. Our aim is to describe differences in reported outcomes for patients born with isolated Hypoplastic Left Heart Syndrome or Transposition of the Great Arteries in a UK population, following either antenatal or postnatal diagnosis.

**Methods:**

Retrospective population-based study with case note review covering a 15 year period (1st January 1998 to 31st December 2012) in the British county of Leicestershire. Cases were identified from two local registers: the East Midlands and South Yorkshire Congenital Anomaly Register and a list of surgical patient held by the East Midlands Congenital Heart Centre.

**Results:**

In total 52 cases of Hypoplastic Left Heart Syndrome or Transposition of the Great Arteries were identified with 24 (46.2%) diagnosed antenatally. Maximum and minimum follow up was 181 and 16 months respectively. Median follow up was 83 months (IQR: 44–111).

The risk of intubation in the postnatal period (OR: 4.64, 95% CI: 1.40 - 15.32) was greater in cases of Hypoplastic Left Heart Syndrome or Transposition of the Great Arteries diagnosed after birth when compared to those diagnosed antenatally. There was a non-significant increase in the risk of metabolic acidosis in the postnatal period (OR: 12.5, 95% CI: 0.64 - 245.46). No differences in mortality or long-term outcomes were demonstrated between antenatally and postnatally diagnosed cohorts.

**Conclusions:**

These results confirm data from American and European populations that, for a British population, an antenatal diagnosis of a major congenital heart disease can have a favourable impact on some postnatal outcome measures. There appears to be no evidence that time of diagnosis impacts on long-term outcome measures.

## Background

In the UK, one in 180 children is born with Congenital Heart Disease (CHD) [1]. Diagnosis can be made antenatally - something which has become increasingly common since the routine implementation of 20-week fetal anomaly scanning and the development of modern ultrasonography [2] - or following symptom presentation in the post natal period.

It has been suggested that short term outcomes in congenital heart disease would improve with antenatal diagnosis because birth in an appropriate setting can be planned in terms of the timing of delivery and immediate access to specialist staff and facilities [3]. In addition, those neonates without an antenatal diagnosis are at risk of discharge without their condition being identified, despite routine baby checks. Where the child is affected by major congenital heart disease that is essentially unstable, this can lead to haemodynamic compromise and result in emergency admission. Similarly, access to surgical intervention may be delayed, or else undertaken with the patient in a sub-optimal condition [4].

Despite this sound reasoning, and whilst improvements in some postnatal measures with an antenatal diagnosis have been demonstrated [5-8], such benefits have not been identified consistently. Much of the existing literature focuses on reviews that are based within tertiary care centres, thus limiting cases to a highly selective group with the greatest potential to survive. There is little reporting of longer term outcomes and sparse data concerning a relevant British population.

### Objectives

The aim of the study was to explore the effect of antenatal diagnosis on congenital heart disease mortality and long term morbidity within a British population.

The specific objectives where to:

- Investigate if an antenatal diagnosis of transposition of the great arteries (TGA) or Hypoplastic left heart syndrome (HLHS) results in improved short term outcomes, i.e. neonatal mortality, risk of metabolic acidosis, Apgar score and need for intubation in the perinatal period.
- Investigate if an antenatal diagnosis of TGA or HLHS correlated with an improvement in long term outcomes, i.e. late mortality, neurodevelopmental deficits, ongoing symptoms and reliance on medication.

## Methods

### Study design

This study comprised a retrospective analysis of outcome data in patients diagnosed with Hypoplastic Left Heart Syndrome or Transposition of the Great Arteries whose mothers lived within the British county of Leicestershire, which has an annual birth rate of approximately 11,000 per year. From 2010 all pregnant women were offered fetal anomaly scanning at around 20 weeks of gestation. From 1998 to 2010 such scanning was offered to a significant proportion of women but was not universal.

Cases were identified from two separate sources: the East Midlands and South Yorkshire congenital anomalies register (EMSYCAR) and the cardiac surgical database held by the East Midlands Congenital Heart Centre (EMCHC). This cross validation of cases was used to ensure complete ascertainment and facilitate accuracy regarding the epidemiology of major congenital heart disease in Leicestershire. A standardised data collection proforma was used to gather information from the medical records.

Isolated Hypoplastic Left Heart Syndrome (HLHS) and Transposition of the Great Arteries (TGA) were chosen for investigation based on their relatively high case numbers locally, case homogeneity and existing literature suggesting a significant proportion of cases were diagnosed antenatally in pregnancies subject to fetal anomaly scanning.

### Setting

All cases of Hypoplastic Left Heart Syndrome and Transposition of the Great Arteries born within the county of Leicestershire between 1st January 1998 and 31st December 2012 were identified. A case note review was then attempted. Identification was primarily through the East Midlands and South Yorkshire Congenital Anomalies Register [9]. This database collects data on all congenital anomalies diagnosed across its constituent counties and is one of a network of 12 regional/disease specific registers that make up the British Isles Network of Congenital Anomaly Registers (BINOCAR) [10]. A database of surgical patients held by the local tertiary care centre (East Midlands Congenital Heart Centre, EMCHC) was used for validation and identification of additional cases.

### Participants

A search of the EMSYCAR database was undertaken for diagnoses of ICD-10 Q234 (HLHS) and Q203 (TGA) born in the study period. Cases were limited to those with a Leicestershire postcode at birth.

A separate list of cases was created from the database of surgical patients held by the EMCHC (Glenfield Hospital, Leicester) for the same period. Clinical notes were then reviewed and relevant data extracted. This comprised clinical information only to ensure anonymity and to comply with information governance; no patient identifiers were recorded.

Within the existing literature there is inconsistency in the approach to cases with a chromosomal diagnosis in terms of inclusion/exclusion [11-13]. Ultimately it was decided to exclude all cases with a confirmed chromosomal defect in order to provide a clearer picture of the implications of antenatal diagnosis for isolated defects.

Cases were also excluded if found to have additional cardiac defects outside those typically seen with diagnoses of HLHS or TGA. A ventricular septal defect in a case of TGA (a common association) was not an exclusion criterion. Cases with major extra-cardiac malformations likely to impact on morbidity or mortality were also excluded.

### Variables

Data points collected were grouped into three life stages: the birth and postnatal period, the surgical period and the post-surgical period (see Table 1). Items within the surgical period group were incomplete for some cases due to intervention in hospitals outside of the East Midlands Congenital Heart Centre. All data were as documented in the clinical case notes.

**Table 1:** **Collected data items**

Birth/Postnatal period	
Birth status	Stillborn, Liveborn, Liveborn later died
Gestational age	Rounded to the nearest week
Delivery method	Vaginal, Assisted Vaginal, Elective C-S, Emergency C-S
Apgar @ 5 mins	
Acidosis in postnatal period	pH < 7.35 on any Arterial Blood Gas within postnatal period
Intubation	Needed mechanical ventilation prior to surgery
Surgical period	
Listed	Yes or No. If no, reason: i.e. Parental or MDT choice
Weight at time of surgery	*Grammes*
Age at time of surgery	*Days*
Cardiopulmonary bypass time	*Minutes*
Cross-clamp time	*Minutes*
Length of post-op ICU stay	*Hours* if <4 days; *days* if >4 days
Surgical mortality	Mortality between induction and post-op ICU discharge.
Post-surgical period	
Ongoing cardiac symptoms	Adjudged at max follow-up. E.g. SOB, decreased exercise tolerance
Ongoing cardiac medications	Adjudged at max follow-up. E.g. β-blockers, Diuretics
Need for re-intervention	
Neurodevelopmental impairment	Referral to specialist service (e.g. SALT) or specific diagnosis (e.g. Cerebral Palsy of Developmental delay)
Mortality	

Summary of the data items collected for each case, split into the postnatal period, surgical period and the post-surgical/long-term period.

SOB: Shortness of Breath. SALT: Speech and Language Therapist.

### Study size

As a local population-based study, it was limited by the number of cases of HLHS and TGA born within the available time-frame of 15 years, the period of data collection for the EMSYCAR database.

### Statistical methods

Data were collated and analysed with IBM SPSS Statistics 20. Antenatal detection rates for each condition were compared to the 50% target set by the Fetal Anomaly Screening Programme (FASP) [14]. Comparisons between the outcomes detected in the antenatal and postnatal cohorts were made, with odds ratios and 95% confidence intervals calculated. Where data items were missing from the notes, cases were excluded from the specific outcome analysis between cohorts.

### Ethics

Ethics permission was provided by the Trent research ethics committee in relation to the East Midlands and South Yorkshire Congenital Anomalies Register.

## Results

### Participants

**Figure 1:**
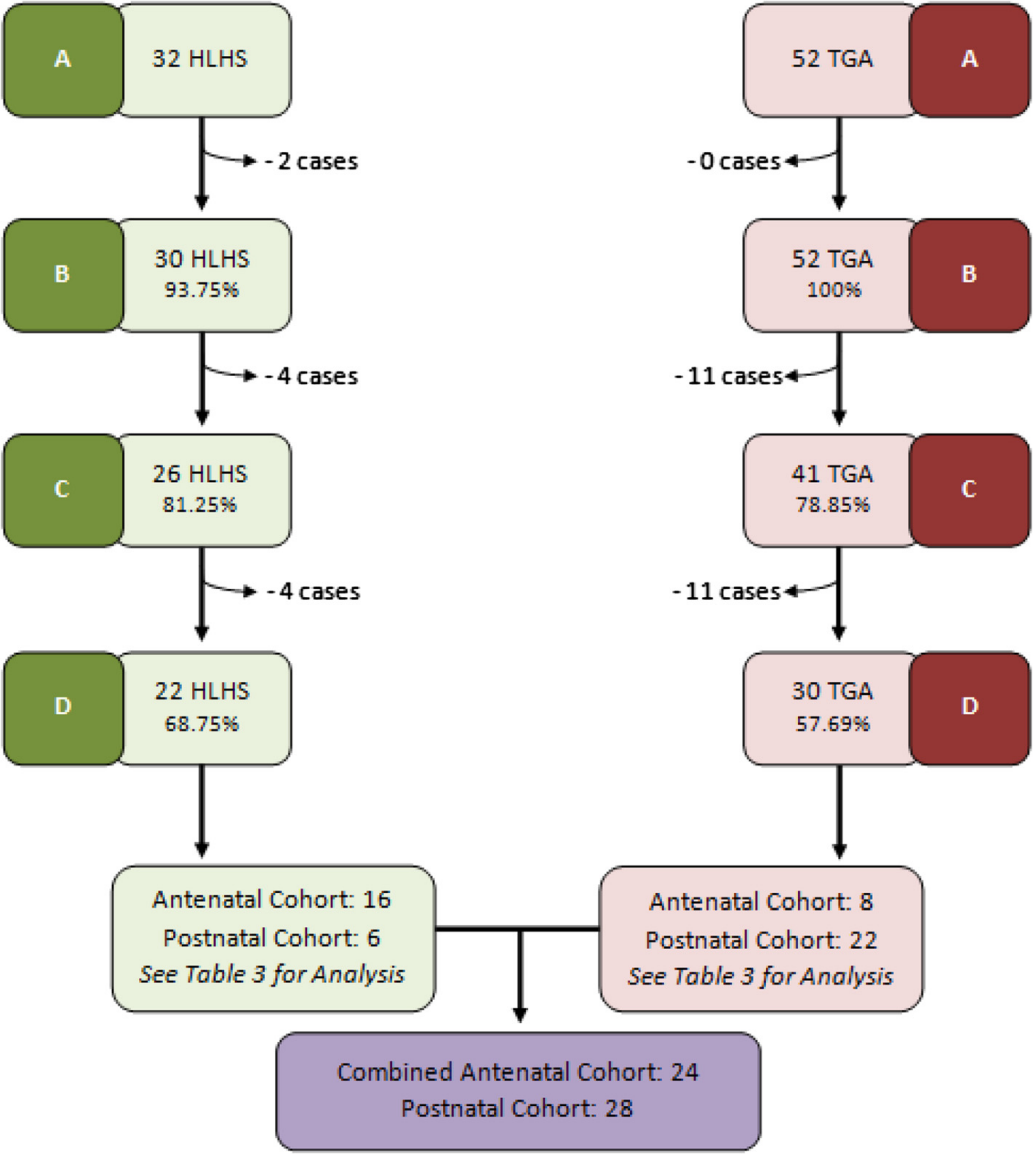
Summary of case inclusion/exclusion. Summary of case numbers of TGA and HLHS and those lost due to exclusion. Antenatal and postnatal cohorts were compared for each diagnosis, then collectively. **A**: Number of cases identified from EMSYCAR and EMCHC database searches. **B**: All cases minus those excluded due to change of diagnosis at birth. **C**: Minus those excluded due to chromosomal diagnosis. **D**: Minus those excluded due to extra-cardiac manifestations or cardiac complexity.

Search of both databases identified 32 HLHS and 52 TGA cases. Two of the HLHS cases identified from EMSYCAR were later downgraded to milder cardiac defects (Shone’s Complex and Patent Foramen Ovale with Ventricular Septal Defect) and were excluded from analysis. Four HLHS and 11 TGA cases were excluded due to an association with a chromosomal diagnosis or for concurrent cardiac defects (see Figure 1).

At case note review, a further 15 cases (Four HLHS and 11 TGA) had to be excluded as insufficient clinical data could be accessed. Total cases used in the final analysis numbered 22 for HLHS and 30 for TGA. Perinatal outcome analysis was also carried out for both conditions combined. This was based on all 52 cases; 24 in the antenatally detected cohort and 28 in the postnatally detected cohort (see Figure 1).

### Descriptive data

Case characteristics are outlined in Table 2. The maximum and minimum follow up periods were 181 and 16 months respectively. Median follow up was 83 months (IQR: 44–111).

**Table 2:** **Patient characteristics by diagnosis**

	**HLHS (Ant)**	**HLHS (Post)**	**OR (95% CI) or p-value**
Number of cases	16	6	
Antenatally diagnosed, %	72.7		
Gender:			
Female, n(%)	11 (68.8)	2 (33.3)	
Male, n(%)	5 (31.2)	4 (66.7)	
Median birth weight, g(IQR)	3042 (2720–3392)	3780 (3490–4100)	p = 0.08
Median gestational age, weeks(IQR)	39 (38–39)	40.5 (38–42)	p = 0.17
C-Section, n(%)	4 (25.0)	2 (33.3)	1.50 (0.20 - 11.54)
Received surgical intervention, n(%)	15 (93.8)	5 (83.3)	3.00 (0.16 - 57.37)
	**TGA (Ant)**	**TGA (Post)**	**OR (95% CI) or p-value**
Number of cases	8	22	
Antenatally diagnosed, %	36.4		
Gender:			
Female, n(%)	1(12.5)	2 (9.1)	
Male, n(%)	7 (87.5)	20 (90.9)	
Median birth weight, g(IQR)	3525 (3415–3870)	3400 (3200–3600)	p = 0.49
Median gestational age, weeks(IQR)	39 (38–41)	40 (38.5 - 41)	p = 0.37
C-section, n(%)	1 (12.5)	3 (13.6)	1.10 (0.10 - 12.47)
Received surgical intervention, n(%)	8 (100)	21 (95.5)	1.19 (0.04 - 32.08)

This table summarises the case characteristics for HLHS and TGA patients by diagnostic stage. There were no significant differences in birth parameters between the antenatal and postnatal cohort. The strong predominance for Male Transposition of the Great Arteries cases is unexpected and unexplained.

In the HLHS group nearly three quarters were antenatally diagnosed. There were no significant differences between the birth weight or gestational age in the antenatally and postnatally detected cohorts and all but one case for each cohort had surgery. For the TGA group, two thirds were postnatally diagnosed and there were no significant differences in birth weight or gestational age between the antenatally and postnatally diagnosed cohorts.

### Outcome data

Outcome measures for the antenatal and postnatal cohorts of HLHS and TGA are presented in Table 3 and Table 4.

**Table 3:** **Outcome measures for antenatally, postnatally diagnosed HLHS**

**Hypoplastic left heart syndrome**		**Antenatal diagnosis**	**Postnatal diagnosis**	**OR (95% CI) or p-value**
Liveborn cases		16	6	
Case status				
Liveborn - alive:		10	5	
Liveborn - later died:		6	1	
Postnatal	Median Apgar Score	9	9	n/a
	Pre-operative Acidosis, *n(%)*	12 (75.0)	6 (100.0)	4.68 (0.21 - 100.99)
	Intubation, *n(%)*	5 (31.3)	5 (83.3)	11 (1.00 - 120.44)
	Mortality within first week, *n(%)*	0 (−)	1 (16.7)	n/a
Surgical	Attempted Surgery, *n*	15	5	n/a
	Median Age at Surgery (IQR), *days*	3.5 (3–7.5)	7 (5–21.5)	p = 0.16
	Median Weight at Surgery (IQR), *gram*	3060 (2900–3510)	3900 (2360–4200)	n/a
	Median Bypass Time (IQR), *mins*	153 (123.5 - 192.5)	134 (117–151)	n/a
	Length of ICU Stay Post Surgery, *days*	16.5 (7–27.5)	11 (8–14.5)	n/a
Long-term outcome	30 day survival (all Liveborn)	75.0% (12/16)	83.3% (5/6)	0.60 (0.05 - 6.80)
	1 Year survival (all Liveborn)	62.5% (10/16)	83.3% (5/6)	0.33 (0.31 - 3.58)
	3 Year survival (all Liveborn <2010)	76.9% (10/13)	80.0% (4/5)	0.83 (0.66 - 10.60)
	5 Year survival (all Liveborn <2008)	75.0% (6/8)	75.0% (3/4)	n/a
	10 Year Survival (all Liveborn <2003)	- (0/0)	50.0% (1/2)	n/a
	Documented neurodevelopment issues, *n*	4/10	2/5	p = 1.00
	Cases requiring on-going cardiac medication, *n*	9/9	4/4	n/a
	Cases with ongoing cardiac symptoms (All), *n*	8/10	1/4	0.08 (0.01 - 1.29)
	Cases with ongoing cardiac symp. (Fontan), *n*	3/4	1/3	n/a

Comparison between outcomes seen in patients with antenatally and postnatally detected Hypoplastic Left Heart Syndrome over three life periods: postnatal period, surgical period and long-term.

Denominators for long-term outcomes decreased as cases were lost from analysis due to absence of clinical information.

**Table 4:** **Outcome measures for antenatally, postnatally diagnosed TGA**

**Transposition of the great arteries**		**Antenatal diagnosis**	**Postnatal diagnosis**	**OR (95% CI) or p-value**
Liveborn cases		8	22	
Case status				
Liveborn - alive:		8	19	
Liveborn - later died:		0	3	
Postnatal	Median Apgar Score *(IQR)*	9 (8–9)	9 (9–9)	n/a
	Pre-operative Acidosis, *n(%)*	8 (100.0)	22 (100.0)	n/a
	Intubation, *n(%)*	1 (12.5)	12 (54.5)	8.40 (0.88 - 80.27)
	Mortality within first week, *n(%)*	0 (0.0)	1(4.5)	1.19 (0.04 - 32.08)
Surgical	Attempted Surgery	8	21	n/a
	Median Age at Surgery (IQR), *days*	12.5 (6–26)	29.5 (10–29)	p = 0.39
	Median Weight at Surgery (IQR), *gram*	3900 (3400–3920)	3450 (3200–4205)	p = 0.40
	Median Bypass Time (IQR), *mins*	170 (144–227)	189 (177–269)	p = 0.50
	Length of ICU Stay Post Surgery, *days*	5 (3.5 - 19)	8 (5–8)	p = 0.89
	Post-Op Complication, *n(%)*	2 / 8 (25.0)	2 / 21 (9.5)	0.32 (0.32 - 2.75)
Long-term outcome	30 day survival (all Liveborn)	100% (8/8)	90.9% (20/22)	2.07 (0.09 - 47.90)
	1 Year survival (all Liveborn)	100% (8/8)	86.3% (19/22)	3.05 (0.14 - 65.78)
	3 Year survival (all Liveborn <2010)	100% (6/6)	83.3% (15/18)	2.94 (0.13 - 65.26)
	5 Year survival (all Liveborn <2008)	100% (5/5)	85.7% (12/14)	2.20 (0.09 - 53.85)
	10 Year Survival (all Liveborn <2003)	100% (2/2)	83.3% (5/6)	n/a
	Documented neurodevelopment issues	1/8	3/19	1.31 (0.12 - 14.93)
	Cases with post-surgical cardiac symptoms	1/8	3/15	1.75 (0.15 - 20.23)
	Cases requiring cardiac medication	2/8	3/15	0.75 (0.10 - 5.77)

Comparison between outcomes seen in patients with antenatally and postnatally detected Transposition of the Great Arteries over three life periods: postnatal period, surgical period and long-term.

Denominators for long-term outcomes decreased as cases were lost from analysis due to absence of clinical information.

For HLHS, the major differences between the two cohorts were in the postnatal measures. Of the antenatal cohort, 75% had a recorded metabolic acidosis compared to 100% in the postnatal cohort - OR: 4.68 (95% CI: 0.21 - 100.99). The antenatal cohort was less likely to require intubation and mechanical ventilation in the postnatal period but due to the small numbers the odds ratio did not quite reach significance: 31.3% vs. 83.3% - OR: 11 (95% CI: 1.00 - 120.44). Assessment of the surgical measures was difficult for the HLHS group as many patients received surgery outside of the local tertiary paediatric cardiac care centre. Neither cohort experienced significantly better long-term outcome measures such as ongoing symptoms (Antenatal: 80%, Postnatal: 25% - OR: 0.08 (95% CI: 0.01 - 1.29)), neurodevelopmental issues (Antenatal: 20%, Postnatal: 20%) or long-term mortality (see Table 3).

For the TGA group, there was also a difference in the numbers requiring intubation in the postnatal period. Intubation was documented in just one case (12.5%) of antenatally diagnosed neonates with TGA, compared to 12 (54.5%) of those diagnosed postnatally - OR: 8.40 (95% CI: 0.88 - 80.27). Surgical measures could be assessed for this group, but no difference in age at surgery (p = 0.39), weight at surgery (p = 0.40) or length of post-op ICU stay (p = 0.89) was found. Similarly there were no differences in long-term outcome measures between the two cohorts.

When the two diagnoses were combined (see Table 5), there was a significant difference in the risk of requiring intubation for those diagnosed postnatally - OR: 4.64 (95% CI: 1.40 - 15.32). There was also an increased risk of metabolic acidosis - OR: 12.5 (95% CI: 0.64 - 245.46) but this was not significant. The only patients to die within the first week of life across both diagnoses were diagnosed postnatally.

**Table 5:** **Outcome measures for anteatally, postnatally diagnosed isolated HLHS or TGA**

		**Antenatal diagnosis**	**Postnatal diagnosis**	**OR (95% CI) or p-value**
Liveborn cases		24	28	
Case status				
Liveborn - alive:		18	24	
Liveborn - later died:		6	4	
Short term	Median Apgar Score	9	9	*
	Pre-operative Acidosis, *n(%)*	20/24 (83.3)	28/28 (100.0)	12.5 (0.64 - 245.46)
	Intubation, *n(%)*	6/24 (25.0)	17/28 (60.7)	4.64 (1.40 - 15.32)
	Mortality within first week, *n(%)*	0/24 (−)	2/28 (7.1)	4.62 (0.21 - 101.16)
	30 day survival	83.3% (20/24)	89.3% (25/28)	0.60 (0.12 - 3.00)
	1 Year survival	75.0% (18/24)	85.7% (24/28)	0.50 (0.12 0 2.04)

Comparison between cases antenatally and postnatally diagnosed with isolated HLHS or TGA. Short term outcomes only were included due to the appropriateness of analysing long-term outcomes for a disparate group.

## Discussion

### Key results

This study has highlighted two specific outcomes where an antenatal diagnosis is favourable - risk of metabolic acidosis and need for postnatal intubation. The finding is seen most clearly in the combined cohorts and this approach of combining the effect on differing anomalies has been reported previously in the American and European literature for both outcomes [7,8]. Our findings suggest that antenatal diagnosis of HLHS and TGA in the UK results in improved perinatal outcomes. However in relation to the increased risk of intubation in the postnatally diagnosed cohort this could reflect a more cautious approach to treatment in this patient group where, at presentation, there is often a range of diagnostic possibilities. Conversely, where a provisional diagnosis already exists treating clinicians may be prepared to adopt a more conservative approach to management. We noted no difference in later outcomes or in terms of overall survival when comparing antenatally and postnatally diagnosed cases.

### Interpretation

The impact of antenatal diagnosis on some perinatal outcomes has been reported previously. Fuchs et al. [5] investigated >250 patients over a 10 year period across four different diagnoses (including TGA), with an 83% antenatal detection rate. The maximum follow up period was 130 months, with a median of 37.8 months. In the prenatally diagnosed cohort, evidence of pre-operative cardiac failure, need for re-surgery and duration of post-operative ICU stay were all found to be significantly favourable (p-value ≤ 0.05) compared to those diagnosed postnatally. Long-term, they found no difference in dependency on cardiac medication or long term survival between the two groups. However, being based in a tertiary care centre, they were unable to include patients without a prenatal diagnosis who died before they were transferred to their unit.

Calderon et al. [6] also explored some longer term outcomes, specifically the effect of prenatal detection of TGA on neurocognitive development. This prospective study showed a correlation between postnatal diagnosis and a less favourable score in “cognitive flexibility” and “social cognition”. Both results were statistically significant and highlighted the need for further investigation in terms of how congenital heart disease can impact on neurodevelopment.

Levey et al. [15] looked at a wider range of congenital heart diseases. Their four year retrospective review included 439 cases of varying diagnoses, creating a large heterogeneous group with widely disparate outcomes. They highlighted a reduced risk of preoperative intubation (OR 0.62, p = 0.033) and emergency surgery (OR 0.18, p < 0.001) in the prenatally diagnosed cohort. No differences were demonstrated in mortality or neonatal measures such as Apgar score or time to surgery.

### Limitations

This study aimed to overcome the potential bias of similar papers, identifying cases from a geographically defined population rather than just those referred to a single tertiary centre. In this way, postnatally diagnosed cases that did not undergo surgery were included. Despite this, surgical data could not be gathered for those operated on outside the East Midlands Congenital Heart Centre, which could limit the interpretation of some of the long term outcomes gathered.

The EMSYCAR database relies on case notification from medical professions. Multiple notifications per case are encouraged to ensure high levels of ascertainment, but some degree of incomplete referral is likely. As fetal detection is one key point for referral, the postnatally diagnosed cohorts are the most likely groups to have been subject to such an affect.

### External validity

All cases were Leicestershire born. The county has a strong reputation for the treatment of congenital heart disease which may or may not be comparable to other areas of the UK. The impact of this local specialist service on parental decision making regarding maintenance of an antenatally diagnosed CHD pregnancy is unknown. The balance of antenatal and postnatal cohorts may have been affected by this.

### Bias

With respect to the neuro-developmental outcomes, a potential source of bias is the increased follow-up afforded to some cases. It is possible that the cases which are more medically unstable (and therefore have more frequent reviews) will be more likely to have a negative outcome diagnosed and recorded if present, than those cases that are reviewed less frequently.

The study utilised two separate patient registers to overcome the potential selection bias of other papers with a similar focus, i.e. those based in a specialist centre which focused only on cases which underwent surgery. The majority of cases were already present on both databases, suggesting high levels of reporting.

## Conclusion

This small study, based on a geographically defined population, has been able to confirm the findings of previous case series of apparent short term benefits of antenatal over post natal diagnosis. No differences in longer term outcomes were identified and this too is in keeping with previous case series. However both the nature of previous studies and the relatively small number of patients reviewed here means that any effect on long term outcome is uncertain. Rates of adverse neurodevelopmental outcome in babies with major congenital heart disease remain relatively high and merit further investigation to understand fully whether antenatal diagnosis has the potential to improve longer term outcome.

## Abbreviations

BINOCAR: British Isles Network of Congenital Anomaly Registers
CHD: Congenital heart disease
EMCHC: East Midlands Congenital Heart Centre, Glenfield Hospital, Leicester, UK
EMSYCAR: East Midlands and South Yorkshire Congenital Anomaly Register
HLHS: Hypoplastic left heart syndrome
TGA: Transposition of the Great Arteries

## References

[1] Townsend N, Bhatnagar P, Wickramasinghe K, Williams J. Children and young people statistics. 2013. 2013;2013. https://www.bhf.org.uk/~/media/files/research/heart-statistics/g694-bhf-children-and-young-people-statistics-2013.pdf.

[2] NICOR. National Institute for Cardiovascular Outcomes Research. [https://nicor4.nicor.org.uk/CHD/an_paeds.nsf/vwContent/Antenatal%20Diagnosis?Opendocument]

[3] Brown KL, Sullivan ID (2014). Prenatal detection for major congenital heart disease: a key process measure for congenital heart networks. Heart.

[4] Brown KL, Ridout DA, Hoskote A, Verhulst L, Ricci M, Bull C (2006). Delayed diagnosis of congenital heart disease worsens preoperative condition and outcome of surgery in neonates. Heart.

[5] Fuchs IB, Muller H, Abdul-Khaliq H, Harder T, Dudenhausen JW, Henrich W (2007). Immediate and long-term outcomes in children with prenatal diagnosis of selected isolated congenital heart defects. Ultrasound Obstet Gynecol.

[6] Calderon J, Angeard N, Moutier S, Plumet MH, Jambaque I, Bonnet D (2012). Impact of prenatal diagnosis on neurocognitive outcomes in children with transposition of the great arteries. J Pediatr.

[7] Kumar RK, Newburger JW, Gauvreau K, Kamenir SA, Hornberger LK (1999). Comparison of outcome when hypoplastic left heart syndrome and transposition of the great arteries are diagnosed prenatally versus when diagnosis of these two conditions is made only postnatally. Am J Cardiol.

[8] Landis B, Levey A, Levasseur S, Glickstein J, Kleinman C, Simpson L, Williams I (2013). Prenatal diagnosis of congenital heart disease and birth outcomes. Pediatr Cardiol.

[9] University of Leicester E. EMSYCAR - East Midlands & South Yorkshire Congenital Anomalies Register. [http://www2.le.ac.uk/departments/health-sciences/research/timms/projects/car]

[10] BINOCAR. British Isles Network of Congenital Anomaly Registers. [http://www.binocar.org/]

[11] Masuda M, Kado H, Tanoue Y, Fukae K, Onzuka T, Shiokawa Y, Shirota T, Yasui H (2005). Does Down syndrome affect the long-term results of complete atrioventricular septal defect when the defect is repaired during the first year of life?. Eur J Cardiothorac Surg.

[12] Formigari R, Michielon G, Digilio MC, Piacentini G, Carotti A, Giardini A, Di Donato RM, Marino B (2009). Genetic syndromes and congenital heart defects: how is surgical management affected?. Eur J Cardiothorac Surg.

[13] Morris CD, Magilke D, Reller M (1992). Down’s syndrome affects results of surgical correction of complete atrioventricular canal. Pediatr Cardiol.

[14] Donna Kirwan, NHS Fetal Anomaly Screening Programme. 18^+0^ to 20^+6^ Weeks Fetal Anomaly Scan National Standards and Guidance for England. 2010.

[15] Levey A, Glickstein JS, Kleinman CS, Levasseur SM, Chen J, Gersony WM, Williams IA (2010). The impact of prenatal diagnosis of complex congenital heart disease on neonatal outcomes. Pediatr Cardiol.

